# Colonoscopic image synthesis with generative adversarial network for enhanced detection of sessile serrated lesions using convolutional neural network

**DOI:** 10.1038/s41598-021-04247-y

**Published:** 2022-01-07

**Authors:** Dan Yoon, Hyoun-Joong Kong, Byeong Soo Kim, Woo Sang Cho, Jung Chan Lee, Minwoo Cho, Min Hyuk Lim, Sun Young Yang, Seon Hee Lim, Jooyoung Lee, Ji Hyun Song, Goh Eun Chung, Ji Min Choi, Hae Yeon Kang, Jung Ho Bae, Sungwan Kim

**Affiliations:** 1 Interdisciplinary Program in Bioengineering, Graduate School, Seoul National University, Seoul, 08826 South Korea; 2 Transdisciplinary Department of Medicine and Advanced Technology, Seoul National University Hospital, Seoul, 03080 South Korea; 3 Department of Biomedical Engineering, Seoul National University College of Medicine, Seoul, 03080 South Korea; 4 Medical Big Data Research Center, Seoul National University College of Medicine, Seoul, 03080 South Korea; 5 Artificial Intelligence Institute, Seoul National University, Seoul, 08826 South Korea; 6 Institute of Medical and Biological Engineering, Medical Research Center, Seoul National University, Seoul, 03080 South Korea; 7 Institute of Bioengineering, Seoul National University, Seoul, 08826 South Korea; 8 Biomedical Research Institute, Seoul National University Hospital, Seoul, 03080 South Korea; 9 Department of Internal Medicine and Healthcare Research Institute, Healthcare System Gangnam Center, Seoul National University Hospital, Seoul, 06236 South Korea

**Keywords:** Colonoscopy, Biomedical engineering, Medical imaging

## Abstract

Computer-aided detection (CADe) systems have been actively researched for polyp detection in colonoscopy. To be an effective system, it is important to detect additional polyps that may be easily missed by endoscopists. Sessile serrated lesions (SSLs) are a precursor to colorectal cancer with a relatively higher miss rate, owing to their flat and subtle morphology. Colonoscopy CADe systems could help endoscopists; however, the current systems exhibit a very low performance for detecting SSLs. We propose a polyp detection system that reflects the morphological characteristics of SSLs to detect unrecognized or easily missed polyps. To develop a well-trained system with imbalanced polyp data, a generative adversarial network (GAN) was used to synthesize high-resolution whole endoscopic images, including SSL. Quantitative and qualitative evaluations on GAN-synthesized images ensure that synthetic images are realistic and include SSL endoscopic features. Moreover, traditional augmentation methods were used to compare the efficacy of the GAN augmentation method. The CADe system augmented with GAN synthesized images showed a 17.5% improvement in sensitivity on SSLs. Consequently, we verified the potential of the GAN to synthesize high-resolution images with endoscopic features and the proposed system was found to be effective in detecting easily missed polyps during a colonoscopy.

## Introduction

Colorectal cancer (CRC) is the third most common cancer diagnosed globally^1^. A screening colonoscopy is the proven modality that enables a reduction in CRC risk through early detection and removal of premalignant colorectal polyps^2^. The two main types of precancerous lesions of CRC are conventional adenomas (ADs, the precursors of 70% of all CRCs) and sessile serrated lesions (SSLs, the precursors of 15–30% of all CRCs)^3^. The detection rate of premalignant polyps is the key quality indicator in a colonoscopy^4^. However, the overall detection rate has varied significantly among individual endoscopists, owing to different recognition skills and the withdrawal technique^5^. Additionally, risk factors leading to missed polyps, such as flat or sessile shapes, a pale color, and small size, can affect the detection rate^6^. Previous studies reported a wide variation in the adenoma detection rate (9.4–32.7%) and SSL detection rate (0.6–11%) across endoscopists, and the overall polyp miss rate is approximately 22%^5,7,8^. The variation of the detection rate and the high polyp miss rate significantly affect the efficacy of colonoscopies for CRC prevention^9^.

Artificial intelligence technology based on deep learning is being applied in various medical fields, and research is being actively conducted to develop computer-aided detection (CADe) systems for colonoscopies to overcome the limitation of the variance of human skills^10–12^. Color, texture, and shape-based features have been used to detect polyps, and polyp detection systems using convolutional neural networks (CNNs) have shown promising results in recent studies^11–14^. A meta-analysis that included five randomized control trials reported that CADe groups exhibited a 44% increase (36.6% vs. 25.2%) in the adenoma detection rate (ADR) and a 70% increase (58% vs. 36%) in the number of ADs per colonoscopy (APCs) when compared with the control groups^15^. These well-trained CADe systems demonstrated high performance for adenoma detection^15–17^. However, the fidelity of CADe systems for SSL detection is still lacking, owing to the very low SSL detection performance when compared with the clinical benchmark for the SSL detection rate (> 5%)^15,18^. SSLs are a high-risk precursor for CRC; however, they can be easily missed even by experienced endoscopists, owing to their subtle morphology with indistinct border, pale color, and flat or sessile shape^19–24^. Large individual variations in the SSL detection rate have also been observed in clinical experts who have been extensively trained to detect SSLs^25^. Therefore, systems specializing in SSL detection must be developed to improve detection performance by detecting additional CRC precursors that may not be visually recognized^26^.

Training data composition is important for a colonoscopy CADe system because colon polyp datasets collected from clinical practice are typically imbalanced^27,28^. Prevalence studies indicate that ADs are approximately eight times more prevalent than SSLs, and each type of polyp exhibits unique endoscopic features^29^. These data imbalance problems can introduce a bias in the training process, thereby decreasing the performance of the CADe system^30^. To address these problems, traditional augmentation methods have been explored to expand minor types of data, including flipping, rotation, scaling, and cropping^31,32^. Recently, generative adversarial networks (GANs) have led active research on medical data synthesis and have been considered for various applications^33–35^. However, in the field of endoscopy, it is difficult to generate whole synthetic images because endoscopy does not have a formal protocol and structure^36^. To overcome this problem, studies have employed GANs to generate endoscopic images that include polyps by synthesizing a normal mucosa background and a lesion patch^37,38^. They showed improved detection performance on gastric cancers and colorectal polyps. Nevertheless, these synthesized images have relatively low quality when compared with actual endoscopic images, and they may not reflect an actual endoscopic environment including polyp features such as color and texture. In the present study, a style-based GAN (StyleGAN) method was adopted to decrease the type imbalance by synthesizing high-resolution endoscopic images, almost indistinguishable from real images and including features of SSLs, proven through a visual Turing test. Then, a polyp detection system contributing to the detection of easily missed polyps was developed based on the validated GAN-synthesized images.

## Results

### SSL image synthesis with GAN

**Figure 1:**
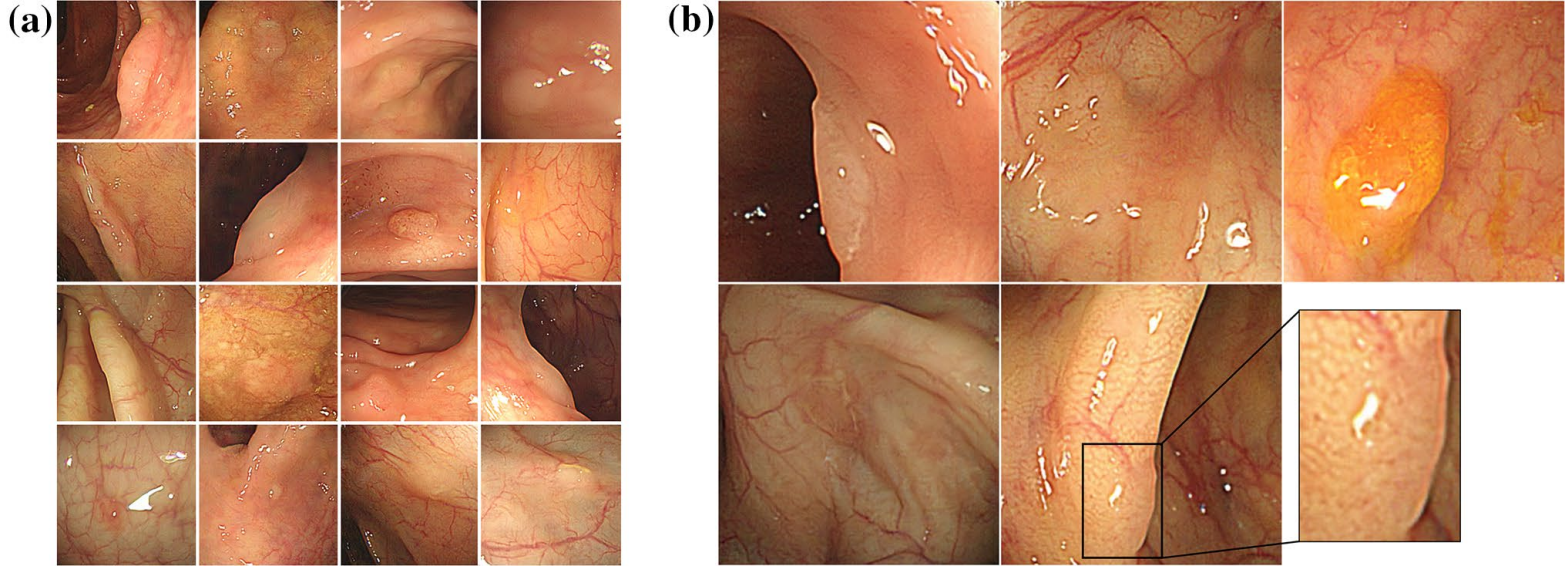
Generated SSL images by StyleGAN2. (**a**) Representative synthetic images with high-quality SSL features, and (**b**) endoscopic features of SSLs discovered in GAN-synthesized SSL images including an indistinct border, flat and irregular shape, mucous cap, a cloud-like surface without vessels, and a dark spot inside the crypts.

In Fig. 1, the synthesized SSL images exhibited global consistency and realistic mucosa features, including texture, color, folds, and blood vessels. Most importantly, the SSLs in the synthesized images depicted the real endoscopic features of SSLs, including sessile or flat morphology, a pale color, disrupted vascular pattern, altered fold contour, indistinct borders with mucus capping, and a rim of bubbles or debris (Fig. 1b). Those features and the quality of synthesized images were identified in the assessment by clinical experts. Additionally, we identified that SSL images with combined features of two or more polyp images present in the GAN training datasets were synthesized (Supplementary Fig. S1). These images can provide more information about the real data distribution. To evaluate the quality of images synthesized with the GAN, we conducted t-SNE visualization and assessments by clinical experts.

#### Fréchet inception distance (FID) score computation

While training StyleGAN2 on the SSL images, we quantified the quality of the synthesized images using an FID score. The FID score was initially 426.0625, while the final FID score was 42.1885 (Supplementary Fig. S2).

#### Visualization of t-SNE

**Figure 2:**
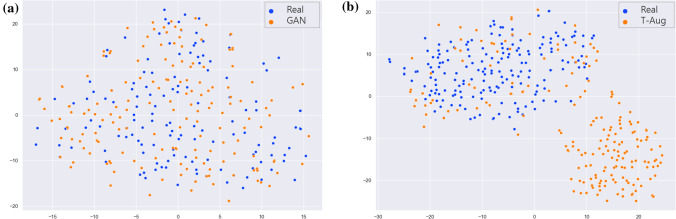
Visualization of SSL augmented images with t-SNE. (**a**) t-SNE of SSL images between original dataset (blue) and GAN augmented dataset (orange), (**b**) t-SNE of SSL images between original dataset (blue) and traditional augmentation dataset (orange).

We used t-SNE visualization to further analyze the synthesized images. The t-SNE algorithm for dimensionality reduction enables the embedding of high-dimensional data into a 2D space. We used t-SNE on a random selection of 200 SSL real images from the original dataset and 200 SSL synthetic images generated by the GAN. As presented in Fig. 2a, the synthetic SSL data group is not separated from the original data distribution, which means that the synthetic images reflected a real distribution. Moreover, it may be helpful to provide more features about the real distribution uncovered by the original dataset. However, we confirmed a discrete distribution between the real images in the original dataset and the traditionally augmented images in Fig. 2b. New features extracted from the traditional augmentation dataset may provide additional information; however, they may not be helpful to approximate the real distribution.

#### Quality assessment with clinical experts

**Figure 3:**
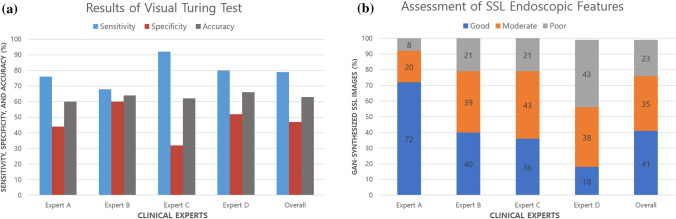
Results of quality assessment with clinical experts. (**a**) Results of a visual Turing test by four experts to differentiate between real and GAN-synthesized SSL colonoscopy images in 1 s, (**b**) Assessment of SSL endoscopic features in 120 representative samples synthesized by the GAN.

We evaluated the image quality of synthetic SSL images with four experts. The prevalence of the samples was blinded to the participating experts. In the first assessment, a visual Turing test was conducted to differentiate between the real and synthetic colonoscopy images; we tested a total of 50 colonoscopy images, which consisted of 25 real and 25 synthetic SSL images that were randomly selected from the real images and GAN-synthesized images. The real images were evaluated as positive, and the synthetic images as negative. All experts showed low performance in identifying whether the lesions shown were true or synthetic. The overall accuracy was 63% (ranging between 60 and 66%), and the sensitivity and specificity for real images was 79% (ranging between 68 and 92%) and 47% (ranging between 32 and 60%), respectively (Fig. 3a, Supplementary Table S1). In the second assessment, a micro assessment of synthetic SSL images was performed according to the characteristic endoscopic features of SSLs, including the indistinct border, pale color, and mucus cap. From the 2400 synthetic SSL images, 120 images were randomly selected for the micro assessment. The image quality was rated in three grades (good, moderate, and poor), representatively shown in Supplementary Fig. S3. The experts judged that 76% (above moderate) of the synthetic SSL images reflected the endoscopic characteristics well (Fig. 3b).

### Performance comparison

#### Performance comparison on polyp detection

To illustrate the validity of the SSL augmentation techniques to improve the detection of premalignant polyps, such as AD and SSL, we evaluated the performance of the augmentation models with the validation dataset, which was composed of 1106 polyp images with 1141 polyps and 1000 normal mucosa images without any polyps. Each polyp image can contain more than one polyp; therefore, the metrics were evaluated per polyp. We implemented the detection models on the whole validation dataset to check the improvement in performance depending on the augmentation techniques and the ratio of the augmented SSLs. To address the imbalance in the organizational ratio of AD, hyperplastic polyps (HP), and SSL within dataset (11:7:1), we augmented SSL images by 1200/1800/2400 corresponding to HP, the average of HP and AD, and AD. As can be observed in Table 1, the models trained on the augmented dataset with two augmentation methods achieved relatively higher performance when compared with the original model. Although the GAN augmentation (GAN-aug) models showed slightly less positive predictive value (PPV) than the traditional augmentation (T-aug) models, they achieved better performance than other models for the average precision (AP) and F1-score, which indicates the overall performance of the detection model. To compare all the augmented models, we focused on AP, which is the representative metric of object detection. Supplementary Fig. S4 shows the variation in the performance (AP) on polyp detection according to the proportion of augmented SSL images in the training dataset in comparison with the original dataset, original + 1200 (aug1), original + 1800 (aug2), and original + 2400 (aug3) augmented datasets. Compared with the performance of the original model, both augmentation methods exhibited a highly improved performance. However, it can be confirmed that data augmentation over a particular proportion causes performance degradation. Through the augmentation methods, we identified that 1800 SSL image generation leads to the best performance in each method. In Table 1, the detection model with the GAN-aug2 dataset achieved the best performance, except for the specificity and PPV. SSL is a polyp type that has inconspicuous features; thus, the increase in SSL images can lead to high sensitivity and low specificity. The ROCs of each of the best models in the original, T-aug, and GAN-aug datasets are shown in Supplementary Fig. S5. In addition, public datasets were evaluated to compare the performance of our model with that of models developed in other studies^31,39,40^. A CNN-based approach CUMED of the MICCAI 2015 challenge on polyp detection^39^ and two optimal models using a faster R-CNN proposed by Shin et al.^31^ were compared on ETIS-LaribPolypDB^41^. A model based on YOLOv2 proposed by Lee et al.^40^ was computed on CVC-ClinicDB^42^. As shown in Supplementary Table S2, the GAN-aug2 model achieved the highest sensitivity value on both datasets: 89.4% on ETIS-LaribPolypDB and 91.0% on CVC-ClinicDB. The detected polyp images obtained using our SSL images, ETIS-LaribPolypDB, and CVC-ClinicDB are shown in Supplementary Fig. S6.

**Table 1:** Performance of original, traditional augmentation (T-aug), and GAN augmentation (GAN-aug) models for the validation dataset including polyp (n = 1141) and negative images (n = 1000).

Evaluation on validation dataset (1141 polyps and 1000 negative images) (95% CI)								
Model	Sensitivity	Specificity	PPV	NPV	F1 score	AP	ACC	AUROC
Original	$$\begin{array}{c}0.9053 \,(0.8883{-} 0.9223)\end{array}$$	$$\begin{array}{c}0.9200 \,(0.9032{-} 0.9368)\end{array}$$	$$\begin{array}{c}0.9417 \,(0.9281{-} 0.9553)\end{array}$$	$$\begin{array}{c}0.8949 \,(0.8759{-} 0.9138)\end{array}$$	$$\begin{array}{c}0.9231 \,(0.9076{-} 0.9387) \end{array}$$	$$\begin{array}{c}0.8909 \,(0.8728{-} 0.9090) \end{array}$$	$$\begin{array}{c}{0.9122} \,(0.9001{-} 0.9243) \end{array}$$	$$\begin{array}{c}{0.90} \,(0.8884{-} 0.9140) \end{array}$$
T-aug1	$$\begin{array}{c}0.9299 \,(0.9151{-} 0.9447) \end{array}$$	$$\begin{array}{c}0.9040 \,(0.8857{-} 0.9223) \end{array}$$	$$\begin{array}{c}0.9654 \,(0.9548{-} 0.9760) \end{array}$$	$$\begin{array}{c}0.9234 \,(0.9066{-} 0.9399) \end{array}$$	$$\begin{array}{c}0.9473 \,(0.9343{-} 0.9603) \end{array}$$	$$\begin{array}{c}0.9280 \,(0.9139{-} 0.9430) \end{array}$$	$$\begin{array}{c}{0.9178} \,(0.9060{-} 0.9295) \end{array}$$	$$\begin{array}{c}{0.93} \,(0.9194{-} 0.9411) \end{array}$$
T-aug2	$$\begin{array}{c}0.9377 \,(0.9237{-} 0.9517) \end{array}$$	$$\begin{array}{c}0.9060 \,(0.8891{-} 0.9229) \end{array}$$	$$\begin{array}{c}0.9665 \,(0.9560{-} 0.9769) \end{array}$$	$$\begin{array}{c}0.9273 \,(0.9122{-} 0.9423) \end{array}$$	$$\begin{array}{c}0.9518 \,(0.9394{-} 0.9642) \end{array}$$	$$\begin{array}{c}0.9341 \,(0.9191{-} 0.9345) \end{array}$$	$$\begin{array}{c}{0.9229} \,(0.9115{-} 0.9342) \end{array}$$	$$\begin{array}{c}{0.94} \,(0.9310{-} 0.9511) \end{array}$$
T-aug3	$$\begin{array}{c}0.9343 \,(0.9199{-} 0.9486) \end{array}$$	$$\begin{array}{c}0.9100 \,(0.8922{-} 0.9276)\end{array}$$	$$\begin{array}{c}0.9673 \,(0.9569{-} 0.9738) \end{array}$$	$$\begin{array}{c}0.9239 \,(0.9127{-} 0.9351) \end{array}$$	$$\begin{array}{c}0.9505 \,(0.9379{-} 0.9630) \end{array}$$	$$\begin{array}{c}0.9275 \,(0.9125{-} 0.9423) \end{array}$$	$$\begin{array}{c}{0.9229} \,(0.9116{-} 0.9343) \end{array}$$	$$\begin{array}{c}{0.93} \,(0.9216{-} 0.9431) \end{array}$$
GAN-aug1	$$\begin{array}{c}0.9413 \,(0.9277{-} 0.9549)\end{array}$$	$$\begin{array}{c}0.9030 \,(0.8847{-} 0.9216)\end{array}$$	$$\begin{array}{c}0.9606 \,(0.9493{-} 0.9719)\end{array}$$	$$\begin{array}{c}0.9290 \,(0.9131{-} 0.9449)\end{array}$$	$$\begin{array}{c}0.9509 \,(0.9385{-} 0.9634)\end{array}$$	$$\begin{array}{c}0.9341 \,(0.9197{-} 0.9485)\end{array}$$	$$\begin{array}{c}{0.9234} \,(0.9120{-} 0.9347) \end{array}$$	$$\begin{array}{c}{0.94} \,(0.9346{-} 0.9542) \end{array}$$
GAN-aug2	$$\begin{array}{c}0.9544 \,(0.9371{-} 0.9717)\end{array}$$	$$\begin{array}{c}0.9010 \,(0.8838 {-}0.9177)\end{array}$$	$$\begin{array}{c}0.9511 \,(0.9386{-} 0.9636)\end{array}$$	$$\begin{array}{c}0.9415 \,(0.9279{-} 0.9551)\end{array}$$	$$\begin{array}{c}0.9527 \,(0.9403{-} 0.9650)\end{array}$$	$$\begin{array}{c}0.9450 \,(0.9318{-} 0.9582)\end{array}$$	$$\begin{array}{c}{0.9295} \,(0.9186{-} 0.9404) \end{array}$$	$$\begin{array}{c}{0.96} \,(0.9547{-} 0.9709) \end{array}$$
GAN-aug3	$$\begin{array}{c}0.9448 \,(0.9316{-} 0.9581)\end{array}$$	$$\begin{array}{c}0.8910 \,(0.8717{-} 0.9003)\end{array}$$	$$\begin{array}{c}0.9506 \,(0.9380{-} 0.9632)\end{array}$$	$$\begin{array}{c}0.9340 \,(0.9235{-} 0.9444)\end{array}$$	$$\begin{array}{c}0.9477 \,(0.9383{-} 0.9570)\end{array}$$	$$\begin{array}{c}0.9377 \,(0.9238{-} 0.9517)\end{array}$$	$$\begin{array}{c}{0.9197} \,(0.9081{-} 0.9313) \end{array}$$	$$\begin{array}{c}{0.95} \,(0.9425{-} 0.9609) \end{array}$$

Augmented datasets, which add augmented images 1200/1800/2400 to the original dataset, are each labelled aug1, aug2, and aug3. All models are evaluated with sensitivity, specificity, positive predictive value (PPV), negative predictive value (NPV), F1 score, and average precision (AP), accuracy (ACC), and area under the receiver operating characteristic curve (AUROC).

#### Performance comparison on histological polyp type

To identify the performance improvement in AD and SSL detection, we separated polyps in the validation dataset into three histological types: AD, HP, and SSL. The polyps included in the validation dataset comprised 620 AD, 438 HP, and 63 SSLs. To accurately evaluate the effectiveness of the detection system for SSLs, we collected 130 additional SSL images for the temporal validation dataset between March 2020 and October 2020 from the same institution.

We evaluated three models on the type-separated polyp validation dataset, original model, T-aug2 model, and GAN-aug2 model. Two augmented models were selected because they are representative of the whole validation dataset in each augmentation method. Compared with the original model, polyp detection sensitivity for each of the three types increased for the GAN-aug2 model. Notably, the GAN-aug2 model exhibited a 19.1% sensitivity improvement when compared with the original model on SSL images (Supplementary Table S3).

Because the number of SSLs in the validation dataset was small to assure the effectiveness of the GAN augmentation, we validated with the SSL temporal dataset, which included 130 images with 133 SSLs. As shown in Table 2, by evaluating all models in the SSL temporal validation dataset, we obtained similar results to the augmented models with 1800 SSL images, which showed high performance when compared with the other models. In addition, GAN augmentation methods also exhibited better performance than the traditional augmentation methods in this case. As a result, we can confirm that the GAN is validated for use in augmentation methods. Furthermore, its performance changed according to the identified augmentation ratio, and we could identify the importance of augmentation considering the distribution of the data.

**Table 2:** Sensitivity, positive predictive value (PPV), and average precision (AP) of original model, traditional augmentation models, and GAN augmentation models for sessile serrated lesion (SSL) temporal validation dataset (n = 133).

Evaluation on SSL temporal validation dataset (95% CI)			
Types	SSL (n=133)		
Model	Sensitivity	PPV	AP
Original	0.8421 (0.7802–0.9041)	0.9333 (0.8909–0.9757)	0.8224 (0.7574–0.8875)
T-aug1	0.9023 (0.8518–0.9528)	0.9677 (0.9378–0.9987)	0.8981 (0.8467–0.9495)
T-aug2	0. 9098 (0.8611–0.9585)	0.9603 (0.9271–0.9834)	0.9003 (0.8494–0.9512)
T-aug3	0.8864 (0.8325–0.9403)	0.9669 (0.9365–0.9973)	0.8821 (0.8273–0.9369)
GAN-aug1	0.9242 (0.8774–0.9580)	0.9385 (0.8977–0.9793)	0.9178 (0.8711–0.9645)
GAN-aug2	0.9398 (0.8994–0.9802)	0.9398 (0.8994–0.9802)	0.9302 (0.8869–0.9735)
GAN-aug3	0.9167 (0.8697–0.9637)	0.9528 (0.9168–0.9888)	0.9107 (0.8622–0.9592)

## Discussion

In this paper, we aimed to generate synthetic SSL images using a GAN for data augmentation to address the data imbalance issue that commonly exists in medical image analysis. As observed in Table 3, the distribution of the polyp histology classes is imbalanced, especially for SSLs, which are a high-risk precursor of CRC but are easily missed during a colonoscopy^23,24^. To improve the performance of the polyp detection system on easily missed polyps, data augmentation with GAN-synthesized SSL images was applied. In previous studies, there were some trials to synthesize endoscopy images using a GAN^37,38,43,44^. For domain adaptation and 3D reconstruction through endoscopy images, GAN techniques were used to synthesize intermediate data of the network, which is operated to translate original endoscopy images to another domain^43,44^. Although GAN synthesis studies have also been conducted for detection problems, the methods were bounded to integrate normal mucosa background images and polyp lesion patches^37,38^. These synthesized images through the randomized combinations of lesion patches and normal mucosa have relatively low quality when compared with the actual endoscopic images, and they may not reflect the endoscopic locational information about histological features, such as color, shape of folds according to thickness, and vessel distributions^45–47^. Additionally, only deterministic polyps are generated including limited variations in polyp features in terms of color and texture^38^. To improve detection performance on easily missed polyps, augmented data must contain subtle features of SSLs that are difficult to detect. In our study, StyleGAN2 exhibited the synthesized high-resolution whole endoscopic images including the GAN mixed-style images, which showed the combined features of two or more SSL images. The quality of the synthesized polyp images was evaluated by four expert endoscopists to verify the reality and endoscopic features of the SSLs. The annotation of the synthetic images was checked twice by the operators. The result proved that it is possible to generate high-resolution endoscopy images using a GAN with only polyp images as the input. Moreover, with one histological type, a GAN can generate polyps, including the endoscopic features of each type, such as texture, color, folds, and blood vessels ensuring the reliability of data synthesis with lesion features in the medical domain.

Through the use of these synthetic images, we can achieve high performance, especially on SSLs. Compared with a traditional augmentation method, GAN-aug models exhibited high detection performance overall (Tables 1, 2, and Supplementary Table S3). This revealed that the GAN augmentation technique can be more effective in augmenting endoscopy datasets for detection than traditional augmentation methods. Our results showed that the synthesized polyp images have meaningful features and can be helpful in solving the data imbalance problem in developing detection systems. Moreover, we could identify the reason for the relatively low performance of T-aug models when compared with GAN-aug models through data distribution visualized using t-SNE. In Fig. 2, the augmented images from the GAN are distributed in the same space with real data; however, they may fill in the distributions by adding data points that are not covered by the original dataset. Considering the augmentation number in the training dataset, it is important to consider the ratio of the augmented data according to the composition. We identified that additional augmented data does not lead to improved performance. As shown in Tables 1 and 2, aug2, which augmented the data with 1800 synthetic SSL images, exhibits the best performance. The reason why aug3 does not exhibit an improved performance when compared with aug2 may be that the distribution of 2400 augmented data differs from the actual data distribution. When the generator learns to map a small subset of the possible realistic modes, partial mode collapse occurs^48^. Data augmentation over a certain value with partial mode collapse could distort the data distribution^49,50^.

To develop an effective CADe system, high detection performance for premalignant polyps that are hard to detect, such as SSLs, is the key factor. Zhou et al.^51^ reported an 84.10% sensitivity to SSL frames with a system that achieved an overall sensitivity of 94.07% and 0.98 AUC in a study by Wang et al.^11^. In particular, we could identify a definite difference in detecting SSL polyps between the original model, T-aug model, and GAN-aug model (Supplementary Table S3, Table 2). The GAN-aug2 model achieved a sensitivity of 95.24% and an AP of 0.9338 in the SSL images included in the validation dataset. Additionally, in the SSL temporal validation dataset, we achieved 93.98% sensitivity and 0.9302 AP using the GAN-aug2 model. When performing external validation using public datasets, the GAN-aug2 model also showed the best performance in terms of sensitivity compared with models of other studies^31,39,40^. Using synthesized polyp images with the GAN resulted in a high detection performance for SSLs and was proved to significantly improve the overall polyp detection performance. GAN-synthesized images may approximate the data distribution, which helps the algorithm to be trained in the desired direction.

As a part of future research, we are planning to develop the system with a multi-center dataset and conduct simultaneous video tests to identify the ability of the polyp detection system in comparison with clinical experts. Furthermore, we will synthesize other polyp types, AD and HP, to confirm the effect of augmentation ratio of each type on the detection performance, and thus develop the classification study on polyp type diagnosis using the GAN-synthesized methods. These results can be extended to multi-class detection studies that simultaneously conduct polyp detection and diagnosis. In addition to the GAN augmentation technique, an advanced CADe system can be developed by improving the resolution of endoscopy with real-time image super-resolution and training various parts of polyps through deep neural network correlation learning mechanism^52,53^.

In conclusion, we proposed an enhanced polyp detection system, which demonstrated higher detection performance, especially on SSLs, which are easily missed during a colonoscopy. Moreover, we verified the capacity of GAN in detection problems and confirmed that GAN-synthesized images are realistic and reflect the endoscopic features of polyps. These features can provide additional information and ensure a comprehensive distribution of the non-augmented dataset, which helps the model to achieve the intended direction of training. Furthermore, the endoscopic features of polyp types in GAN-synthesized images could be utilized for histological type diagnosis problems.

## Methods

**Figure 4:**
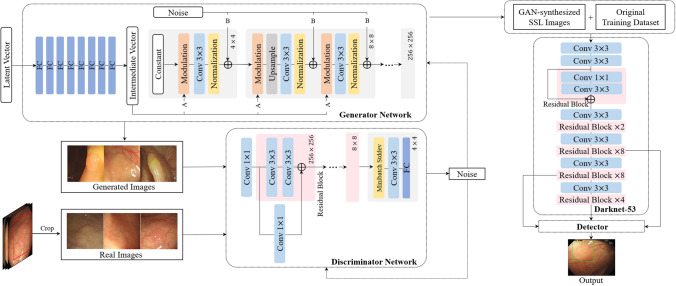
Procedure to develop automatic polyp detection system with SSL augmentation using GAN. In the generator network, “A” means a learned affine transform and “B” operates the noise broadcast.

In this section, we describe the dataset used in developing the polyp detection system, and explain the method for generating synthetic SSL images using GAN and traditional augmentation methods. The quality of the generated SSL images was evaluated using both quantitative and qualitative techniques. To identify the effect of data augmentation depending on the proportion between imbalanced types, SSL minor type augmentations were conducted three times (with an increase in the major type, middle type, and average between those). Then, the performance analysis of the polyp detection models trained with original data, GAN augmentations, and traditional augmentations could be conducted (Fig. 4). The study protocol adhered to the ethical guidelines of the 1975 Declaration of Helsinki and its subsequent revisions, and was approved by Seoul National University Hospital Institutional Review Board (number H-2001-083-1095). A study protocol was designed in consideration of the use of retrospective data of the previous study (number H-1505-019-670), and informed consent from the patients was waived by Seoul National University Hospital Institutional Review Board.

### Dataset

We developed a polyp detection system using 5503 white-light colonoscopy images with polyps from colonoscopy examinations undertaken at the Seoul National University Hospital, Healthcare System Gangnam Center between October 2015 and February 2020. The endoscopic images for the development of the colonoscopy AI system were collected from the retrospective and prospective databases. Retrospective data was collected from the previous study’s database, and informed consent from the patients was waived^54^. Prospective data was collected under written informed consent. All colonoscopies were performed using high-definition colonoscopes (EVIS LUCERA CV260SL/CV290SL, Olympus Medical Systems Co., Ltd., Japan) by nine endoscopists. Polyp images were randomly split in a ratio of 8:2 to compose the training and validation datasets (both images included different sets of polyps). To evaluate negative performance, we used 1000 normal mucosa colonoscopy images with no polyps. In addition, because the number of SSLs in the validation dataset was less than 100, we collected another 130 SSL images in the same process between March and October 2020 for temporal validation. The organizational ratios within the dataset show that ADs, HPs, and SSLs have a ratio of approximately 11:7:1, and SSLs constitute a very small proportion (Table 3). To address this imbalance, we augmented the minor type SSL images by 1200/1800/2400 images corresponding to HP, the average of HP and AD, and AD. Additionally, for external validation, ETIS-LaribPolypDB^41^ and CVC-ClinicDB^42^ were used to evaluate the models. ETIS-LaribPolypDB is composed of 196 images with 208 polyps, and CVC-ClinicDB includes 612 images containing 646 polyps. Details of all the datasets are provided in Supplementary Fig. S7.

**Table 3:** Polyp characteristics for the training, validation, and SSL temporal validation datasets.

		Training dataset	Validation dataset	Temporal validation dataset
The number of images		4397	1106	130
The number of polyps		4423	1141	133
**Polyp characteristics, the number of polyps (%)**				
Histology				
AD		2528 (57%)	620 (56%)	–
SSL		222 (5%)	63 (5%)	133 (100%)
HP		1585 (36%)	438 (37%)	–
Others		88 (2%)	20 (2%)	–

Each image can contain more than one polyp.

*AD* adenoma,* SSL* sessile serrated lesion,* HP* hyperplastic polyp.

### Synthetic SSL image generation

We used two strategies to generate SSL synthetic data: a traditional augmentation technique involving geometric transformations, and synthesized images through a GAN model, which learned using SSL images from the training dataset. In each of the two augmentation methods, three datasets were randomly sampled to a ratio of 1200/1800/2400 depending on the type imbalance. The number of augmented SSL images was determined by the proportional difference between the major (AD), middle (HP), and minor (SSL) types. Datasets to which 1200 synthetic SSL images were added to match the ratio with that of the middle type (HP) are called aug1. Similarly, the other two datasets are called aug2 (1800 images) and aug3 (2400 images), respectively. For performance comparison, we used the same environment and the same hyperparameters.

#### Traditional augmentation method

Data augmentation including geometric transformations, such as rotating, flipping, cropping, and scaling has been traditionally conducted to enlarge the minor type of the training dataset^31,32^. We implemented augmentation techniques on all 222 SSL images in the detector training dataset. Each image was flipped vertically. Then, original non-flipped images and flipped images were rotated three times at random angles $\theta _{1}=[0^{\circ }, \ldots, 120^{\circ }], \theta _{2}=[120^{\circ }, \ldots, 240^{\circ }], \theta _{3}=[240^{\circ }, \ldots, 360^{\circ }]$. Subsequently, the rotated images were randomly cropped to a size of $256\times 256$ pixels twice. Consequently, each image was augmented 12 times $((1+1_{flip})\times 3_{rotation}\times 2_{crop})$; 186 images were removed because they could not be cropped while including the whole polyp region. Then, we sampled the 1200/1800/2400 augmented images randomly to create three traditional augmented datasets, T-aug1, T-aug2, and T-aug3.

#### Image augmentation with GAN

The style-based generator architecture (StyleGAN2) has a hierarchical generator with a skip connection, and it normalizes the convolution weights using estimated statistics instead of normalizing them with actual statistics^55^. It is a promising model for the generation of medical images, owing to its capacity to synthesize high-resolution images with a realistic level of details^56^. The aim of training StyleGAN2 was to synthesize SSL colonoscopy images for improving the CADe performance in polyp detection. The dataset is composed of SSL images, included in the polyp detection training datasets. To train StyleGAN2, 203 SSL images were cropped to a size of $256\times 256$ pixels that included the polyp region; the remaining 19 SSL images were excluded because they could not be cropped to include the polyp. Adam optimizer was used to train StyleGAN2 with momentum parameters $\beta _{1}=0$ and $\beta _{2}=0.99$ at a learning rate of $2\times 10^{-3}$.The main loss function was a non-saturating logistic loss with $R_{1}$ regularization. The training phase required approximately 5625 ticks for a total of 45,000 iterations (54 days) with a batch size of 16 on an NVIDIA GeForce RTX 2080 Ti GPU$\times$ 2 (32 GB RAM). StyleGAN2 proposed an FID score to quantify the quality of the synthesized images every 10 ticks. The generated image resolution was adjusted to $256\times 256 \; pixels$. To verify the differences between the basic image generation model, the image-to-image translation model, and StyleGAN2, we additionally trained DCGAN and CycleGAN on SSL images with the same resolution. After the loss converged, the synthesis results of the three GAN models were compared. As shown in Supplementary Fig. S8, the images synthesized using StyleGAN2 could be confirmed to have the highest resolution and most realistic lesion characteristics. Differences depending on the network type are discussed in the Supplementary Information (Description of the GAN models used).

For quantitative evaluation of the quality of the generated images, we measured the differences between two distributions in the high-dimensional feature space of an Inceptionv3 classifier with FID^57^. If the activations on the real and synthesized data are *N*(*m*, *C*) and $N(m_{w}, C_{w})$ respectively, FID is defined as

1 $$\begin{aligned} \left\| m-m_{w} \right\| _{2}^{2}+Tr(C+C_{w}-2(CC_{w})^{\frac{1}{2}}) \end{aligned}$$

FID was computed with all images in the training dataset and 50,000 random images generated at every 10 ticks. All generated images were annotated with the expected SSL region; those that could not be annotated were discarded. Randomly sampled 1200/1800/2400 synthetic SSL images were added to the original training dataset to compose the GAN-aug1, GAN-aug2, and GAN-aug3 datasets.

#### Assessment of GAN-synthesized images

We used quantitative and qualitative techniques to analyze the results and demonstrate the quality of the synthetic images generated by the GAN^58,59^. In the qualitative evaluation, two methods were applied: (i) t-SNE visualization and (ii) quality assessment by clinical experts. Clinical experts in the quality assessment process were board-certified gastroenterologists with colonoscopy experience ranging from 5 to 20 years, and their annual volume of procedures was over 500. Written informed consent was obtained from all participating physicians.

First, the t-SNE algorithm for dimensionality reduction enabled the embedding of high-dimensional data into a 2D space. It represented the similarities between the data points by iteratively comparing the probability distributions of different data points in both high- and low-dimensional spaces^60^. By applying t-SNE, the distribution of real/synthetic images can be visually analyzed^33,61^. We randomly selected 200 images each from the real-SSL original dataset, traditionally augmented images, and GAN-synthesized images. Then, all images were resized to $224\times 224$ pixels. We set a perplexity of 30 for 1000 iterations.

Second, the synthesized images were evaluated by clinical experts^33,37,43^. Four clinical experts evaluated the visual quality of the SSL samples generated by the GANs. Two test modules were developed using a PowerPoint presentation, and the quality of the evaluation was assessed in two steps: Test 1—visual Turing test: differentiation of a polyp image between a real and GAN-synthesized image within 1 s; Test 2—micro assessment of synthetic SSL image quality (corresponding SSL endoscopic features) in 10% representative SSL synthetic images. Through this process, we aimed to answer the following: (1) Is the appearance of the synthesized lesions realistic? (2) Do the synthesized lesions sufficiently include the characteristic endoscopic features of SSLs? All images used in the tests had a size of $256\times 256$ pixels, and the prevalence of the samples was blinded.

### Training polyp detection model

To develop the real-time polyp detection system, we applied one-stage detection algorithms with high inference speed. Three representative one-stage object detection models, RetinaNet, single shot detector (SSD), and YOLOv3 were trained on the original training dataset with the same environment for performance comparison (Supplementary Table S4). Considering frame per second (FPS) and overall metrics, subsequent experiments were conducted using YOLOv3. Additionally, we confirmed the performance of YOLOv3 according to the backbones used as the feature extractor. Darknet-53 was compared with Inceptionv3, ResNet50, and AlexNet. As YOLOv3 performs detection at three scales, three inputs should be provided to the front model of YOLOv3 in $52\times 52\times 256$, $26\times 26\times 512$, $13\times 13\times 1024$. We placed the outputs from three layers of each model into the front model of YOLOv3 and trained it on the original training dataset with the same parameter settings. As shown in Supplementary Table S5, Darknet-53 achieved a higher performance overall compared to other backbone networks.

Through transfer learning, faster loss convergence can be achieved by initializing the current model with the learned weights of a pre-trained model^62,63^. In this study, a pre-trained Darknet-53 model was used to customize the YOLOv3 detection model. The backbone classifier is the Darknet-53 network, and features were extracted in three different scales for the detection of objects of various sizes^64^ as shown in Supplementary Fig. S9. It uses the sum-squared error between the predictions/ground truth and binary cross-entropy in class prediction as a loss.

2 $$\begin{aligned} &\lambda _{coord}\sum _{i=0}^{S^{2}}\sum _{j=0}^{B}{\mathbb {I}}_{ij}^{obj}(2-w_{i}\times h_{i})[(x_{i}-\hat{x_{i}})^{2}+(y_{i}-\hat{y_{i}})^{2}]\\&\qquad +\lambda _{coord}\sum _{i=0}^{S^{2}}\sum _{j=0}^{B}{\mathbb {I}}_{ij}^{obj}(2-w_{i}\times h_{i})[(w_{i}-\hat{w_{i}})^{2}+(h_{i}\hat{h_{i}})^{2}]\\&\qquad +\lambda _{obj}\sum _{i=0}^{S^{2}}\sum _{j=0}^{B}{\mathbb {I}}_{ij}^{obj}[\hat{C_{i}}log(C_{i})+(1-\hat{C_{i}})log(1-C_{i})]\\&\qquad +\lambda _{noobj}\sum _{i=0}^{S^{2}}\sum _{j=0}^{B}{\mathbb {I}}_{ij}^{noobj}[\hat{C_{i}}log(C_{i})+(1-\hat{C_{i}})log(1-C_{i})]\\&\qquad +\lambda _{class}\sum _{i=0}^{S^{2}}{\mathbb {I}}_{ij}^{obj}\sum _{c\in classes}^{}[\hat{p_{i}}(c)log(p_{i}(c))+(1-\hat{p_{i}}(c))log(1-p_{i}(c))] \end{aligned}$$

where $x_{i}, y_{i}$ is the centroid location of an anchor box, $w_{i}, h_{i}$ are the width/height of the anchor; $C_{i}$ is the objectness, which is the same as the confidence score; and $p_{i}(c)$ is the classification loss. When an object exists in the box*j* of cell*i*, ${\mathbb {I}}_{ij}^{obj}$ is 1; otherwise, it is 0. If the box*j* in cell*i* has no object, the value of ${\mathbb {I}}_{ij}^{noobj}$ is 1; otherwise, it is 0. The size of the feature map is described as $S^{2}$, and *B* is the number of anchor boxes. Lambda coefficients, $\lambda _{coord}, \lambda _{obj}, \lambda _{noobj}$, and $\lambda _{class}$ are the weight parameters for localization, objects, no-object, and classes that were initially set to 1, 5, 1, and 1, respectively, by default. An increase in the lambda coefficients affects the focused part of the loss. Specifically, and increment in $\lambda _{coord}, \lambda _{obj}, \lambda _{noobj}$, and $\lambda _{class}$ affects the intersection over union (IoU), sensitivity, specificity, and confidence score, respectively. We set the lambda coefficients as 1, 9, 2, and 1 based on experimental trials. The model was initialized with ImageNet weights and then fine-tuned to learn the endoscopic features of polyps.

We trained YOLOv3 with a pre-trained Darknet-53 model with a batch size of 8, and the learning rate for the Adam optimizer was $1\times 10^{-7}$, which was selected from the range [$1\times 10^{-4}$ $1\times 10^{-8}$]. The network input size was set to $416\times 416$ such that input images were all resized to $416\times 416$, and nine anchors, which were obtained by clustering the dimensions using K-means for each dataset, were used. Anchors that overlapped the ground truth object by less than the IoU threshold value (0.5) were ignored. The non-maximum suppression threshold value was set to 0.2, and the confidence score was selected as 0.5. More details can be found in the Supplementary Information (Description of training detection algorithm).

The original training dataset is composed of 4397 WL polyp images. All images were added twice to include one with a full monitor image and one that is cropped to involve only the endoscopic view. Additionally, we composed augmentation training datasets with traditional geometric transformation methods and GAN. SSL augmented images were added in 1200/1800/2400 to the original training dataset so that the proportion of SSL in the dataset corresponded to the HP, the average of HP and AD, and AD. To compare the detection performance according to the augmentation methods and augmented ratio of the minor type, we trained the polyp detection model using the same hyperparameters as the original and augmented datasets: (i) original model trained without augmented images; (ii) T-aug1, T-aug2, and T-aug3 models trained with original + 1200/1800/2400 traditionally augmented SSL images; and (iii) GAN-aug1, GAN-aug2, and GAN-aug3 models trained with original + 1200/1800/2400 synthetic SSL images generated by StyleGAN2. We evaluated these models using the original validation dataset and SSL temporal validation dataset, including 130 SSL images with confidence score and IoU thresholds both at 0.5.

### Statistical analysis

In polyp images, the polyp detection box with an IoU higher than 0.5 was evaluated as true-positive (TP) and that lower than 0.5 was evaluated as false-positive (FP). If the system could not detect the polyp box in the polyp images, those images are evaluated as false-negative (FN). To evaluate the instances of a true-negative (TN) result, we composed 1000 negative images with no polyp. Using the TP, FP, FN, and TN indicators, we calculated the sensitivity (= recall), specificity, PPV (= precision), negative predictive value (NPV), AP, F1-score, and the area under the receiver operating characteristic (AUROC).

## Supplementary Information

Supplementary Information.

## Data Availability

The datasets generated during and/or analyzed during the current study are available from the corresponding author on reasonable request.
