# Supplementary material for: Colonoscopic image synthesis with generative adversarial network for enhanced detection of sessile serrated lesions using convolutional neural network
Source: Sci Rep. 2022 Jan 7;12:261. doi: 10.1038/s41598-021-04247-y (PMC8741803; doi:10.1038/s41598-021-04247-y)
Supplement: Supplementary file 1 — Supplementary Information. [file 41598_2021_4247_MOESM1_ESM.docx]

**Supplementary Information**

**Colonoscopic Image Synthesis with Generative Adversarial Network for Enhanced Detection of Sessile Serrated Polyps Using Convolutional Neural Network**

Dan Yoon^1, ⸸^, Hyoun-Joong Kong^2,^^3,4,5, ⸸^, Byeong Soo Kim^1^, Woo Sang Cho^1^, Jung Chan Lee^3,6,7^, Minwoo Cho^2,8^, Min Hyuk Lim^3^, Sun Young Yang^9^, Seon Hee Lim^9^, Jooyoung Lee^9^, Ji Hyun Song^9^, Goh Eun Chung^9^, Ji Min Choi^9^, Hae Yeon Kang^9^, Jung Ho Bae^9,*^ & Sungwan Kim^3,5,7,*^

^1^Interdisciplinary Program in Bioengineering, Graduate School, Seoul National University, Seoul, 08826, Korea

^2^Transdisciplinary Department of Medicine and Advanced Technology, Seoul National University Hospital, Seoul, 03080, Korea

^3^ Department of Biomedical Engineering, Seoul National University College of Medicine, Seoul, 03080, Korea

^4^Medical Big Data Research Center, Seoul National University College of Medicine, Seoul, 03080, Korea

^5^Artificial Intelligence Institute, Seoul National University, Seoul, 08826, Korea

^6^Institute of Medical and Biological Engineering, Medical Research Center, Seoul National University, Seoul, 03080, Korea

^7^Institute of Bioengineering, Seoul National University, Seoul, 08826, Korea

^8^Biomedical Research Institute, Seoul National University Hospital, Seoul, 03080, Korea

^9^Department of Internal Medicine and Healthcare Research Institute, Healthcare System Gangnam Center, Seoul National University Hospital, Seoul, 06236, Korea

Dan Yoon and Hyoun-Joong Kong contributed equally as the first authors.

Jung Ho Bae and Sungwan Kim contributed equally as corresponding authors.

**Correspondence to:**

**Jung Ho Bae, MD**

Department of Internal Medicine and Healthcare Research Institute,

Healthcare System Gangnam Center, Seoul National University Hospital, Seoul 06236, Korea

Email: [newsanapd@naver.com](mailto:newsanapd@naver.com)

**Sungwan Kim, PhD**

Department of Biomedical Engineering, Seoul National University College of Medicine and Institute of Bioengineering, Seoul National University, Seoul 03080, Korea

Email: [sungwan@snu.ac.kr](mailto:sungwan@snu.ac.kr)

Description of training detection algorithm

In the YOLOv3 algorithm with a Darknet-53 structure, a residual module is added to the network, including two convolutional layers and a residual layer. This residual module repeats 1, 2, 8, 8, and 4, and pooling layer is not used. To downsample the feature maps, a convolutional layer with stride 2 is included instead. Leaky ReLU is applied as activation function; other activation functions including ReLU and Mish activation functions were tested. As a result, ReLU showed a slight decrease in performance, and Mish was confirmed to exhibit a significantly poor performance; therefore, we finally used Leaky ReLU as the activation function. The filter, stride size, and overall structure of each layer are shown in Supplementary Fig. S9.

Description of the GAN models used

CycleGAN and DCGAN were compared with StyleGAN by training them on the same dataset. StyleGAN is an image generation model that can synthesize realistic high-resolution images with various styles. Unlike image-to-image translation GAN models, images here are generated through latent style vectors. Therefore, source and reference images are not required for synthesis, and images can be synthesized without limitations. To compare the results of the GANs, we additionally trained DCGAN and CycleGAN. DCGAN is a basic image generation model and CycleGAN is an image-to-image translation model that enables image translation without pair data. As shown in Supplementary Fig. S8, the synthetic images obtained using DCGAN are of low quality, and blurry and crushed features can be observed in images generated using CycleGAN. However, StyleGAN2 synthesized images that included realistic endoscopic features with a high resolution.


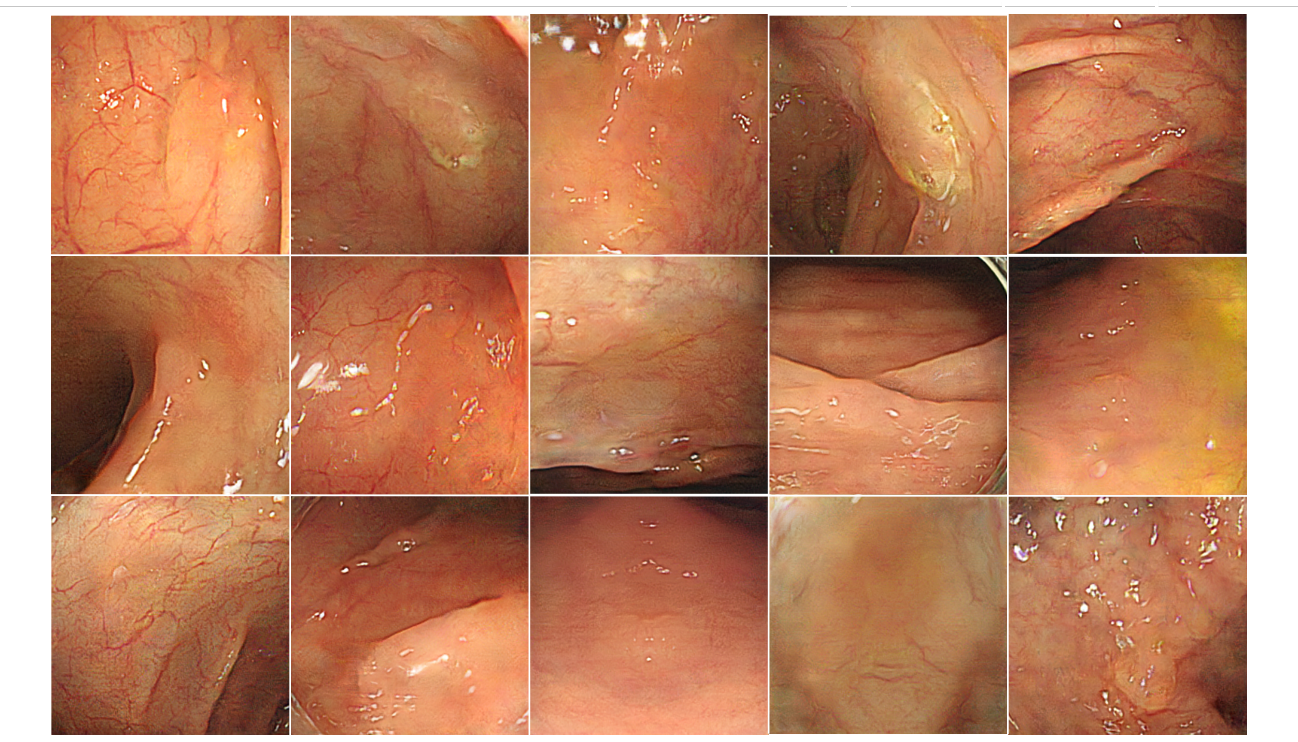


**Supplementary Figure S1.** Mixed styles of synthesized sessile serrated lesion (SSL) images, which show combined features of two or more polyp images through GAN training.


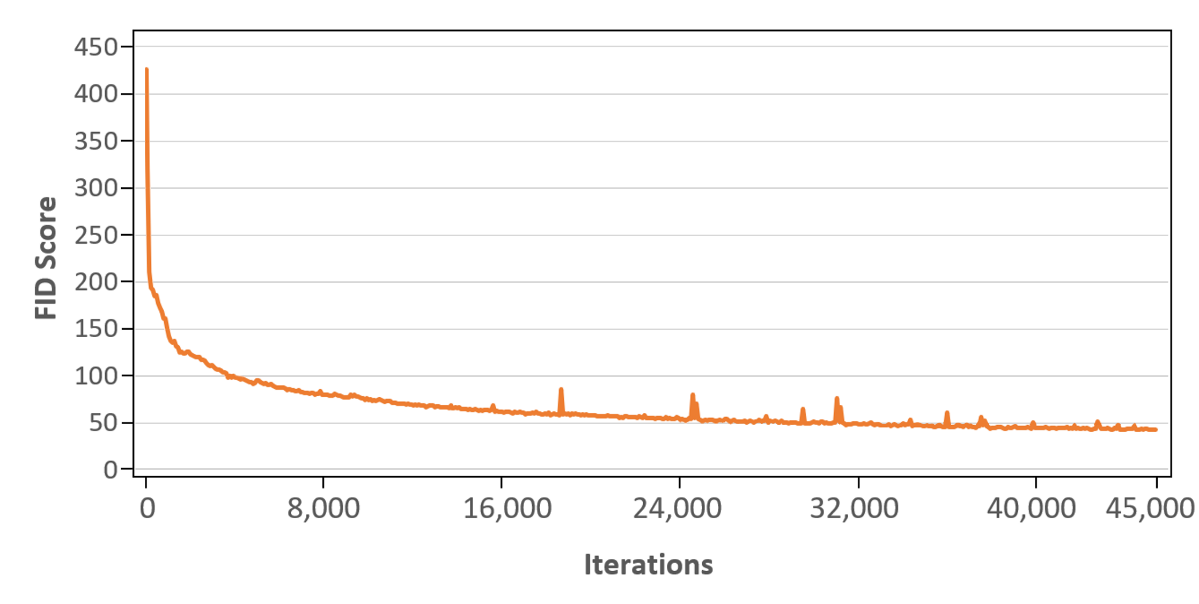


**Supplementary Figure S2.** FID scores for training the GAN on the 203 SSL dataset.


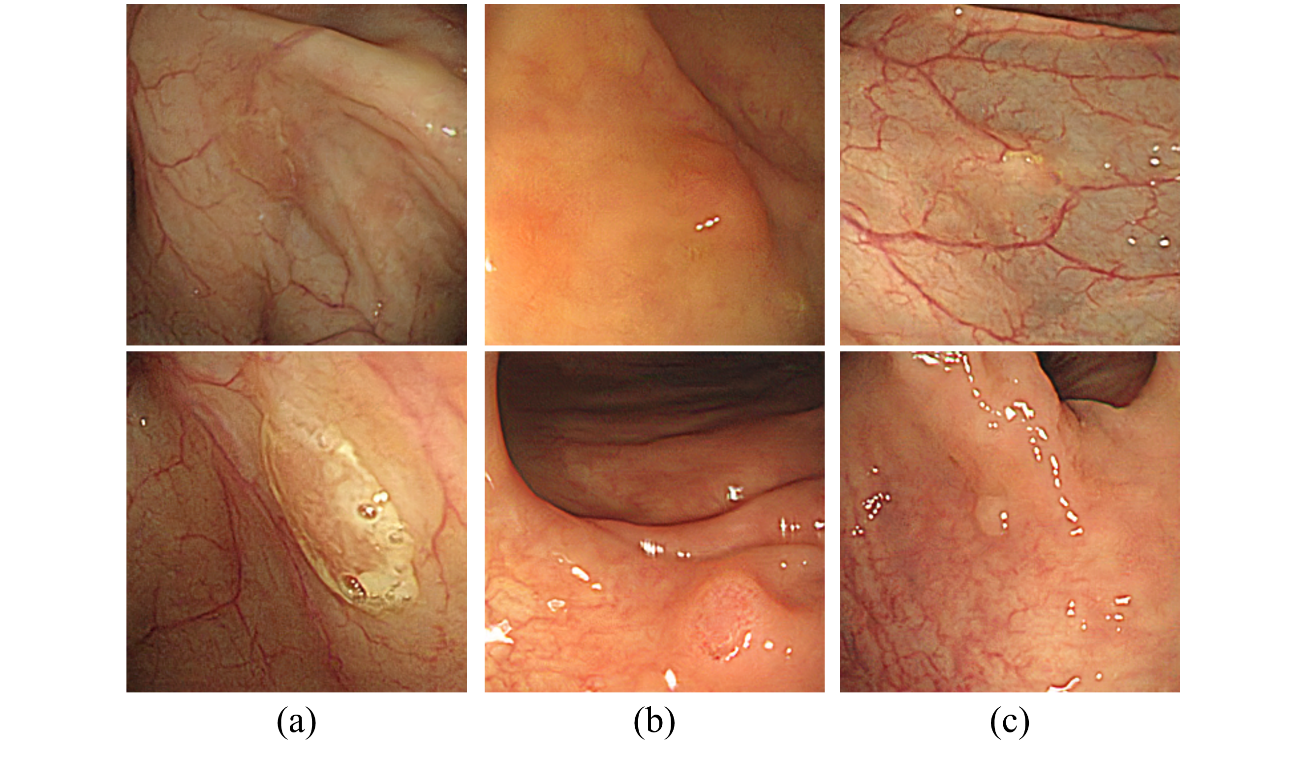
**Supplementary Figure S3.** Representative synthesized SSL images in three grades of a micro assessment on SSL endoscopic features. (a) good images including obvious SSL endoscopic features, (b) moderate images with one or more SSL endoscopic features, and (c) poor images with unclear SSL features.


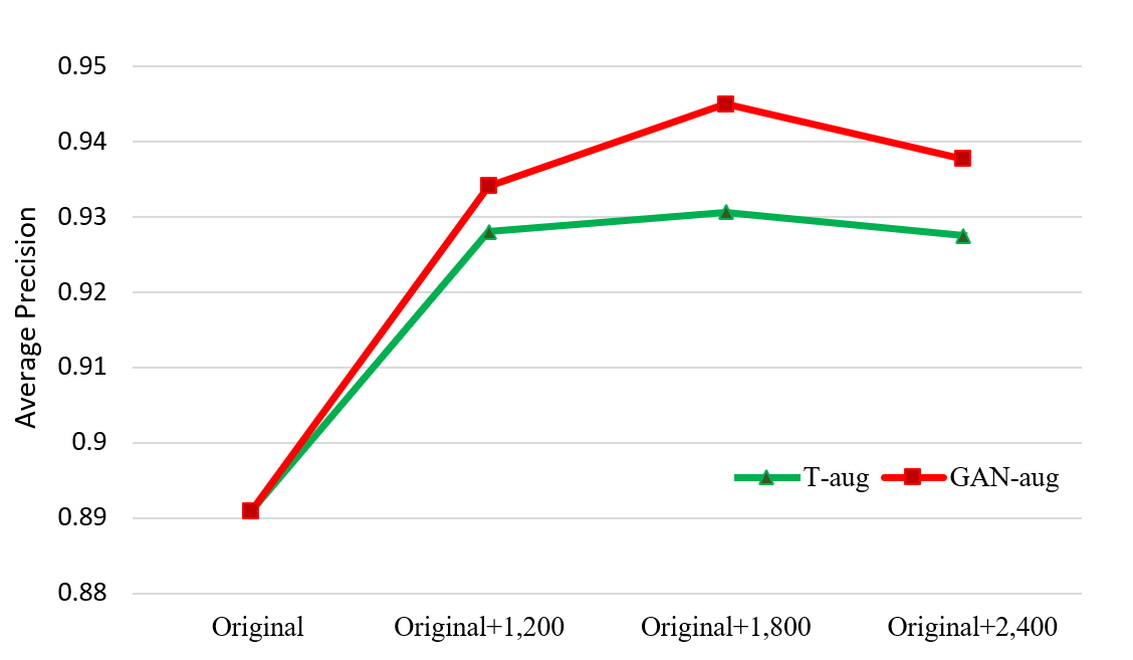


**Supplementary Figure S4.** Variation in average precision (AP) of polyp detection according to the ratio of augmented SSL images.


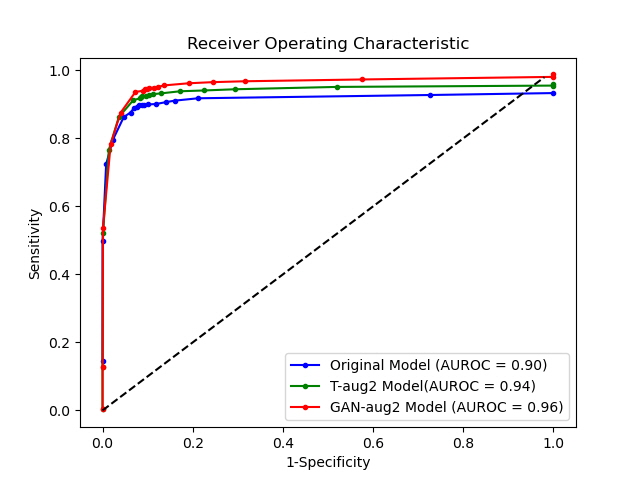


**Supplementary Figure S5.** Receiver operating characteristics (ROCs) of models with original, T-aug2, and GAN-aug2 datasets.

**
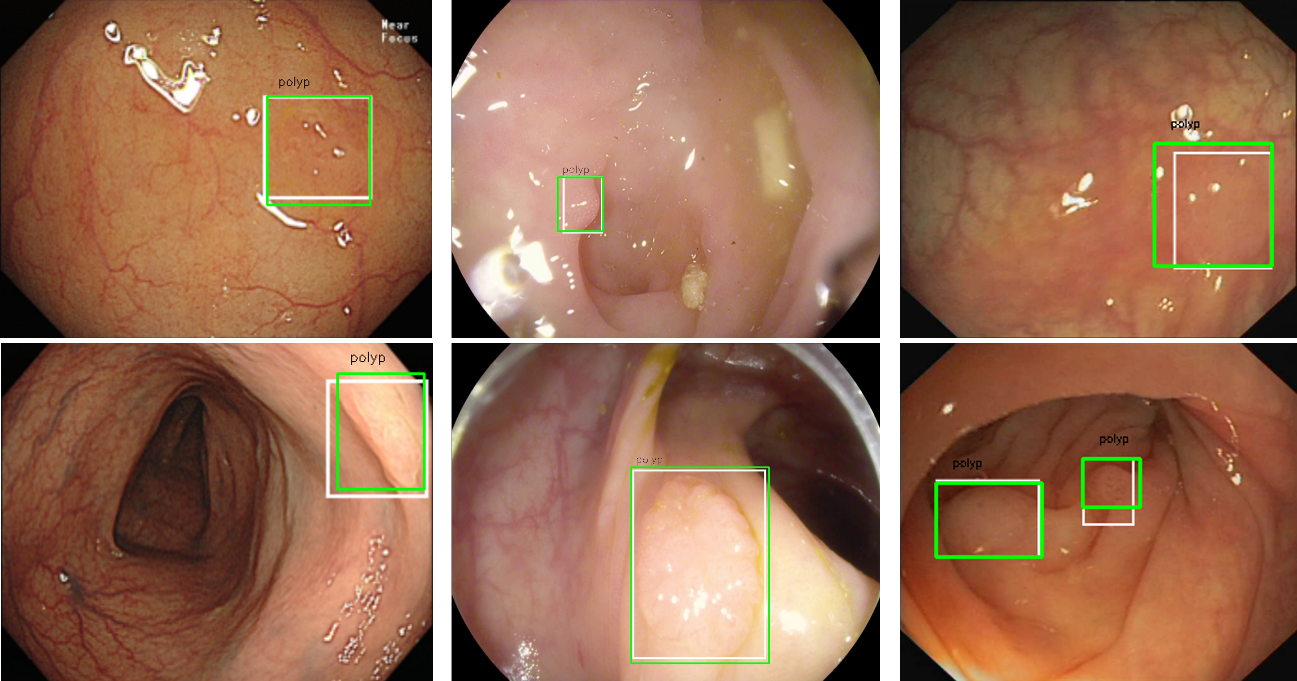
Supplementary Figure S6.** Result images of polyp detection using the GAN-aug2 model (green) marked along with the ground truth (white). Detection results of (a) SSL temporal validation dataset, (b) ETIS-LaribPolypDB, and (c) CVC-ClinicDB.

**Supplementary Figure S7.
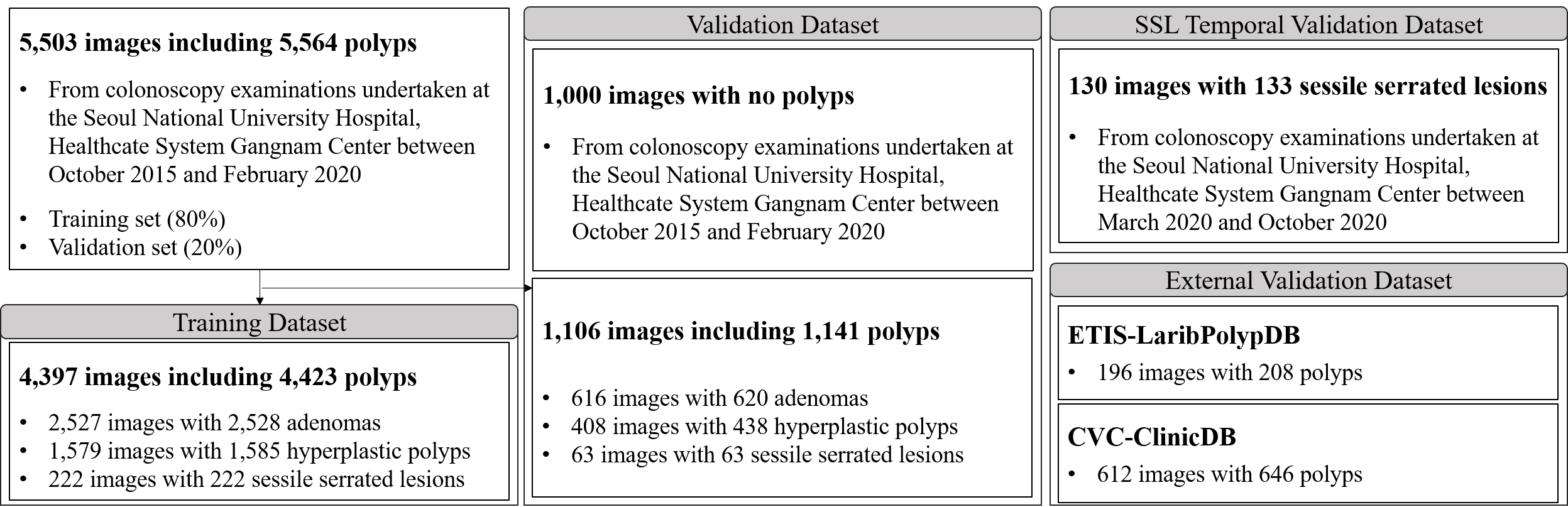
** Detailed contents of the training, validation, SSL temporal validation, and external validation datasets. Each image can contain more than one polyp.

**
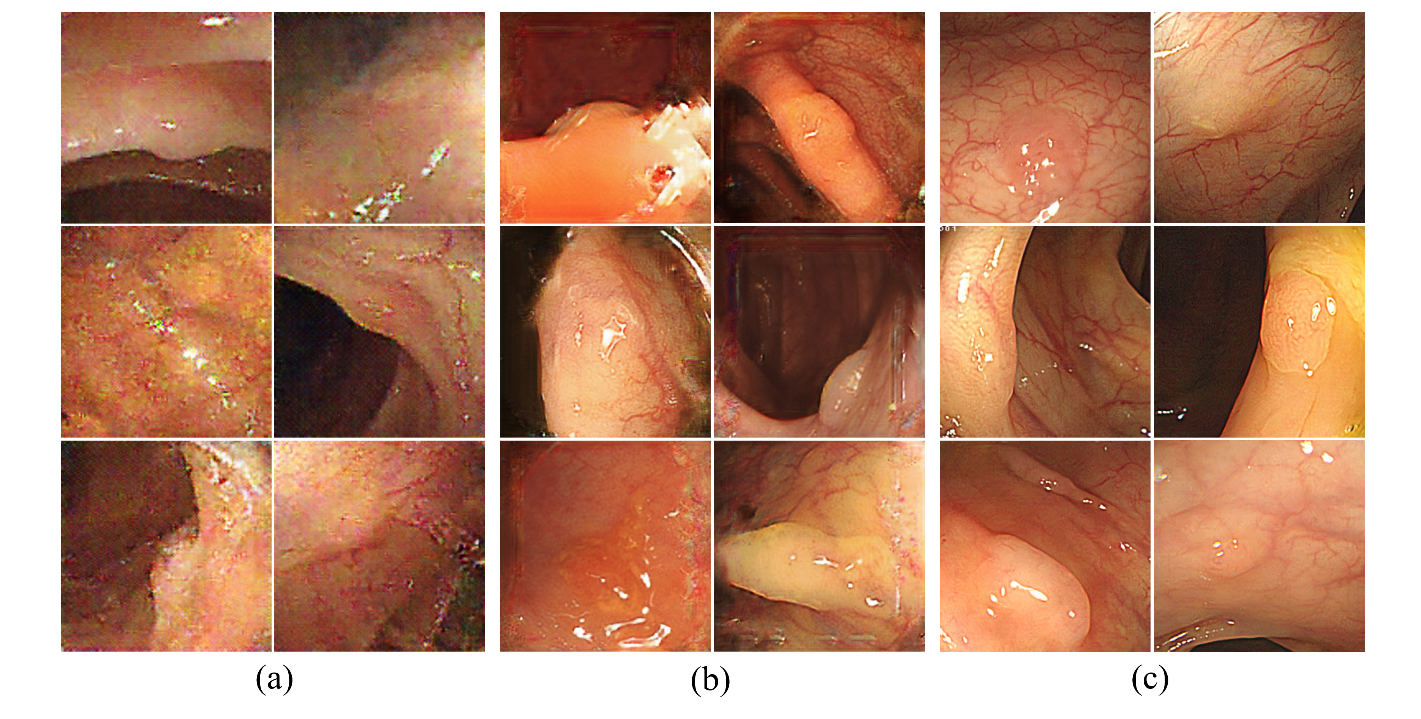
S****upplementary Figure S8.** Quality comparison of the synthesized images. (a) Images generated using DCGAN have a low resolution, (b) images synthesized using CycleGAN have unclear and blurry lesion characteristics, and (c) realistic high-resolution endoscopic features can be observed in images synthesized using StyleGAN2.

**
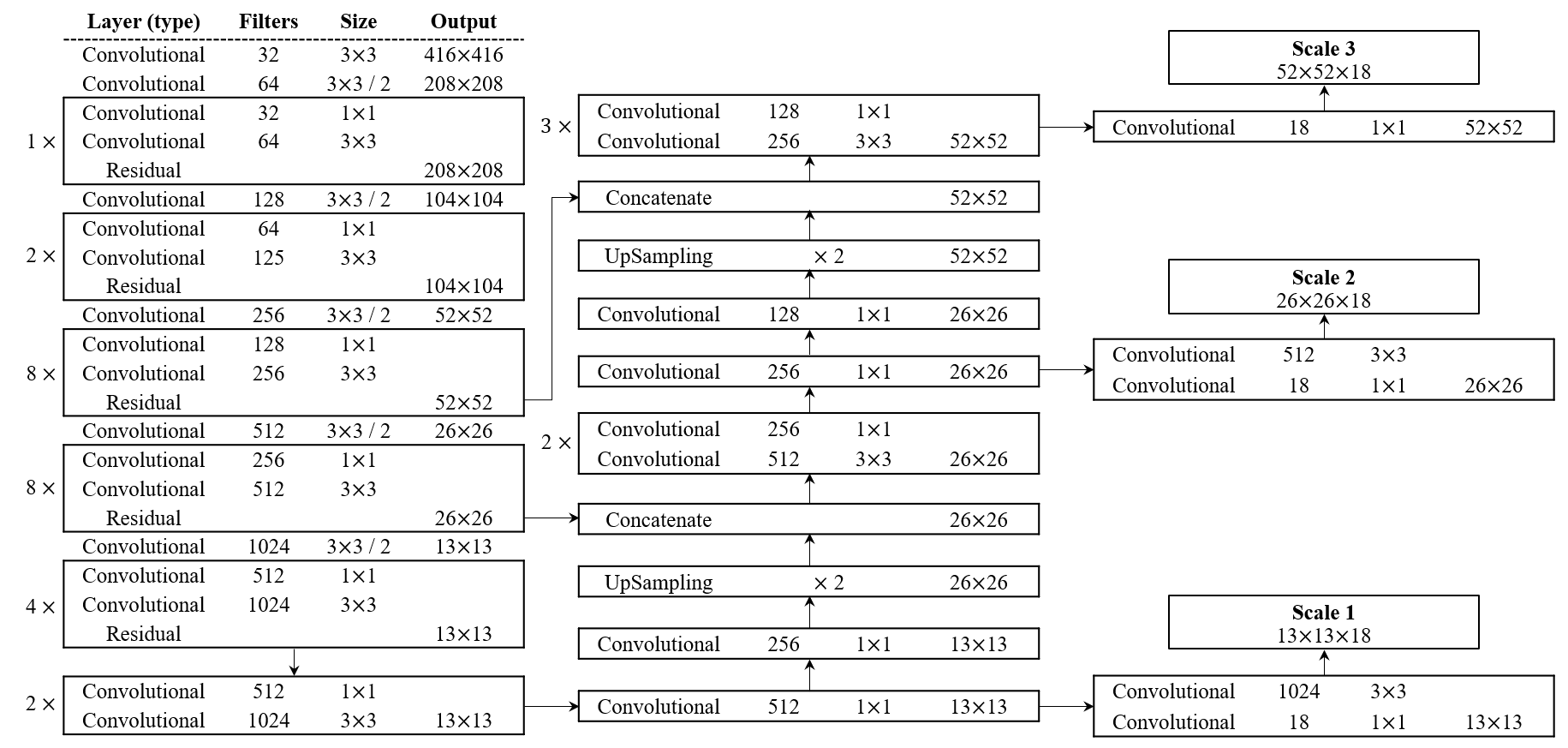
Supplementary Figure S9.** YOLOv3 network architecture used in the training detection model.

**Supplementary Table S1.** Results of a visual Turing test conducted by four experts to differentiate between real and GAN-synthesized sessile serrated polyp colonoscopy images in 1 s. Synt: synthesized; PPV: positive predictive value; NPV: negative predictive value.

| 50 colonoscopy images (25 real + 25 synt) | | | | | |
| --- | --- | --- | --- | --- | --- |
| **Clinical experts** | Accuracy | Sensitivity | Specificity | PPV | NPV |
| Expert A | 0.60 | 0.76 | 0.44 | 0.58 | 0.65 |
| Expert B | 0.64 | 0.68 | 0.60 | 0.63 | 0.65 |
| Expert C | 0.62 | 0.92 | 0.32 | 0.58 | 0.80 |
| Expert D | 0.66 | 0.80 | 0.52 | 0.63 | 0.72 |
| Overall | 0.63 | 0.79 | 0.47 | 0.60 | 0.69 |

**Supplementary Table S2.** Evaluation of original, 1,800 traditional augmentation (T-aug2), and 1,800 GAN augmentation (GAN-aug2) models compared with other studies on the public datasets. Our three models exhibit representative performance on the augmentation methods. All models are evaluated using true-positive (TP), false-positive (FP), false-negative (FN), sensitivity, and positive predictive values (PPVs).

| External Validation Dataset | | | | | | |
| --- | --- | --- | --- | --- | --- | --- |
| **Public Data** | **Model** | TP | FP | FN | Sensitivity | PPV |
| ETIS-LaribPolypDB | CUMED [39] | 144 | 55 | 64 | 0.692 | 0.723 |
|  | Shin et al. [31]  Aug-Ⅰ | 167 | 26 | 41 | 0.803 | 0.865 |
|  | Shin et al. [31]  Aug-Ⅱ | 148 | 14 | 60 | 0.712 | 0.914 |
|  | Original | 165 | 12 | 43 | 0.793 | 0.932 |
|  | T-aug2 | 175 | 23 | 33 | 0.841 | 0.884 |
|  | GAN-aug2 | **186** | 17 | 22 | **0.894** | 0.916 |
| CVC-ClinicDB | Lee et al. [40] | 577 | 10 | 63 | 0.902 | 0.982 |
|  | Original | 534 | 11 | 112 | 0.827 | 0.980 |
|  | T-aug2 | 577 | 14 | 69 | 0.893 | 0.976 |
|  | GAN-aug2 | **588** | 15 | 58 | **0.910** | 0.975 |

**Supplementary Table S3.** Evaluation of original, 1,800 traditional augmentation (T-aug2), and 1,800 GAN augmentation (GAN-aug2) models for histological polyp types. The three models exhibit representative performance on the augmentation methods. AD: adenoma; SSL: sessile serrated lesion; HP: hyperplastic polyp; PPV: positive predictive value.

| Validation dataset | | | | | | |  |
| --- | --- | --- | --- | --- | --- | --- | --- |
|  | AD (n=620) | | SSL (n=63) | | HP (n=438) | |  |
| **Model** | Sensitivity | PPV | Sensitivity | PPV | Sensitivity | PPV | |
| Original | 0.9371 | 0.9635 | 0.7619 | 0.7742 | 0.8995 | 0.9352 | |
| T-aug2 | 0.9661 | 0.9788 | 0.8730 | 0.9483 | 0.9498 | 0.9519 | |
| GAN-aug2 | 0.9806 | 0.9712 | 0.9524 | 0.9231 | 0.9612 | 0.9273 | |

**Supplementary Table S4**. Overall performance of representative one-stage object detection models, RetinaNet, SSD, and YOLOv3. AP: average precision; FPS: frame per second.

| **One-stage detector** | AP | Accuracy | FPS |
| --- | --- | --- | --- |
| RetinaNet | 0.8747 | 0.8170 | 14 |
| SSD | 0.8610 | 0.8707 | 32 |
| YOLOv3 | 0.8909 | 0.9122 | 30 |

**Supplementary Table S5**. Performance of YOLOv3 with representative backbones, Darknet-53, Inceptionv3, ResNet50, and AlexNet. PPV: positive predictive value; AP: average precision.

| Backbone | Sensitivity | PPV | AP |
| --- | --- | --- | --- |
| Darknet-53 | 0.8909 | 0.9122 | 0.8909 |
| Inceptionv3 | 0.8378 | 0.8171 | 0.8238 |
| ResNet50 | 0.8738 | 0.7681 | 0.8476 |
| AlexNet | 0.7301 | 0.8041 | 0.7683 |
